# Study on Residual Stresses and Deformations in Turning of Aerospace Thin-Web Gears Considering the Initial Heat-Treatment State and Clamping Constraints

**DOI:** 10.3390/ma19143039

**Published:** 2026-07-14

**Authors:** Tao Chen, Shengwei Tong, Wenyao Wang, Suyan Li, Wenyuan Xu, Lankui Su, Hao Sun, Catherine Sotova

**Affiliations:** 1 Key Laboratory of Advanced Manufacturing and Intelligent Technology, Ministry of Education, Harbin University of Science and Technology, Harbin 150080, China; 2 School of Mechanical Engineering, Heilongjiang University of Science and Technology, Harbin 150022, China; 3 Harbin Xinhua Aviation Industry Co., Ltd., Harbin 150080, China; 4 Harbin Dongan Engine Co., Ltd., Aero Engine Corporation of China, Harbin 150080, China; 5 Moscow State Technological University STANKIN, Vadkovsky per. 1, Moscow 127994, Russia; e.sotova@stankin.ru

**Keywords:** thin-web gear, machining deformations, final residual stresses, on-machine measurement, clamping force

## Abstract

As transmission systems evolve toward lightweight design, gear webs are becoming thinner and more sensitive to deformation caused by the coupled action of heat-treatment residual stress, finish-turning thermo-mechanical loading, and clamping constraints. Existing studies mainly treat the initial residual stress or the machining-induced residual stress separately and often simplify the clamping boundary as an ideal fixed constraint. To overcome these limitations, this study proposes an initial-field-driven prediction framework for aerospace thin-web gears. The post-heat-treatment residual stress/strain field is reconstructed using the eigenstrain reconstruction method using measured residual stress and deformation data and is then introduced into the ABAQUS finish-turning model as the actual initial state. A three-jaw-chuck boundary consistent with the experiment and a progressive element birth–death strategy driven by the measured cutting force and temperature are used to describe material removal. In addition, a laser displacement sensor on-machine measurement (LOMM) method is developed for initial pose correction, deformation monitoring, and clamping-force interval optimization. The predicted final residual stress (FRS) distribution and machining deformation agree with the experimental measurements, with errors generally below 10%. The optimized clamping-force interval of 1350–1650 N provides a balance between cutting stability and deformation suppression. This work clarifies the coupled roles of the initial heat-treatment state and clamping constraints in thin-web gear finish turning and provides a reproducible modeling route for FRS and deformation prediction.

## 1. Introduction

As helicopters evolve toward higher maneuverability, higher power density, and lower energy consumption, lightweight design of the transmission system has become a key objective for improving overall performance. As the core component of the transmission system, the web of a gear, though not the primary load-bearing area, accounts for the largest proportion of its mass. Therefore, reducing the gear-web thickness has become an effective strategy for structural weight reduction [1]. However, material-removal ratios can reach 80–90% [2,3], which markedly reduces local stiffness and thermal stability. This inevitably causes thin-walled components to undergo shape distortions such as warping and twisting during manufacturing, leading to dimensional deviations. Therefore, how to effectively control residual stress (RS) and machining deformation in thin-walled components has become a critical issue requiring urgent resolution in the field of high-precision manufacturing.

To reveal the RS evolution and deformation mechanisms of thin-walled components during manufacturing, scholars have carried out extensive research. The main methods include experimental methods, analytical methods, and the finite element method (FEM). Experimental methods can measure final residual stress (FRS) and deformation, but they are costly, and it is difficult to reflect the complete evolution process. Analytical methods can theoretically derive the formation mechanisms of RS and deformation, but the model assumptions are overly idealized. In contrast, FEM is currently the most widely applied method for predicting RS and deformation in thin-walled components. It can comprehensively simulate the generation and superposition processes of RS and deformation and obtain the final distribution state [4]. Gao et al. [5] proposed a machining-deformation-prediction model for thin-walled components, showing that the machining deformation is mainly governed by the bending stiffness and the RS distribution characteristics of the components. Cheng et al. [6] and Gang et al. [7] established three-dimensional milling models for Ti-alloy thin-walled components and analyzed the deformation behavior of Ti-alloy materials during milling using FEM. Yi et al. [8] investigated the microscopic deformation mechanism during the milling of thin-walled micro-impeller blades. Xiang et al. [9] investigated the deformation behavior of different types of miniature thin-walled structures during milling, revealing the influence mechanism of dynamic milling forces on thin-walled component deformation. The above studies demonstrate that FEM is an effective method for predicting the RS and deformation of thin-walled components.

During machining processes, machining-induced residual stress (MIRS) is considered one of the primary causes of machining deformation, which is particularly significant in thin-walled components [10]. Existing studies have systematically analyzed the formation mechanism of MIRS from the perspective of processing parameters. Research indicates that the cutting parameters [11,12], multi-pass cutting strategies [13], and tool geometry parameters [14] all significantly influence the distribution pattern and stress levels of MIRS. Yang et al. [15] analyzed the relaxation mechanism of surface MIRS under the influence of CryoMQL cooling technology. Jiang et al. [16] quantitatively analyzed the effects of the cutting force and cutting temperature on MIRS, and the results indicated that the cutting force plays a dominant role in influencing MIRS. Compared to the MIRS generated during the machining process, the initial residual stress (IRS) undergoes complex redistribution during material removal and is also one of the key factors contributing to deformation in thin-walled components [17,18,19]. Yue et al. [20] proposed that IRS dominates early deformation, while MIRS gradually takes over as part stiffness decreases with continuous material removal. Ma et al. [21] introduced IRS into the cutting process and found that IRS significantly affect the cutting force and cutting temperature. Yang et al. [22] investigated the influence of blank IRS on the machining deformation of the overall structure, indicating that IRS is the primary factor affecting machining deformation. Tok et al. [23] investigated the effects of different cutting conditions on RS in heat-treatment components. Ma et al. [24] demonstrated, through FEM and experiments that IRS is the direct source of machining deformation. Saleem et al. [25] employed FEM to analyze the redistribution of IRS as material is removed. Therefore, IRS is considered a critical factor in improving the prediction accuracy of machining deformation in thin-walled components.

In addition, the role of clamping constraints in thin-walled machining cannot be neglected. The proper control of the clamping force is regarded as one of the core elements for ensuring machining quality [26]. When the clamping force is insufficient, the workpiece is highly prone to slippage and vibration during machining [27], which not only undermines machining stability but also leads to dimensional deviations. Conversely, when the clamping force is excessive, it may cause the stress on the part to exceed its yield strength [28], resulting in irreversible plastic deformation. Meanwhile, the clamping force can also couple with IRS/MIRS to induce machining deformation [29]. Therefore, the optimized control of the clamping force and calibration of initial clamping deviations have become critical issues. On-machine measurement technology enables the in situ inspection of workpieces, allowing the timely adjustment of machining parameters and clamping force to shorten manufacturing cycles while promptly detecting machining errors [30,31,32,33].

In summary, existing prediction models for the machining deformation of thin-walled components still have three major limitations. First, many studies focus on either MIRS or IRS, whereas the spatially non-uniform residual stress/strain field generated by carburizing–press quenching is rarely reconstructed and inherited as the actual initial state for finish turning. Second, the boundary condition is commonly simplified as an ideal fixed constraint, which cannot reflect the contact and deformation characteristics of a three-jaw chuck during thin-web gear turning. Third, an on-machine measurement is often used only for dimensional inspection, and its coupling with clamping-force optimization and simulation validation has not been fully established. Compared with previous studies on residual stress, machining deformation, FEM prediction, and clamping-force effects, the main contributions of this work are as follows: (1) a post-heat-treatment residual stress/strain field reconstruction strategy is introduced to provide a physically consistent initial state for finish-turning simulation; (2) a finish-turning FEM model is constructed with experimentally consistent three-jaw clamping and measured thermo–mechanical loads, avoiding the excessive simplification of fixed boundaries; (3) an LOMM-based pose-correction and clamping-force-optimization procedure is developed to support in-situ deformation evaluation; and (4) the coupled evolution mechanism among IRS, MIRS, clamping stress, and material-removal-induced stiffness reduction is discussed for the web–hub transition region of aerospace thin-web gears.

## 2. Construction of the Finish-Turning-Simulation Model

**Figure 1:**
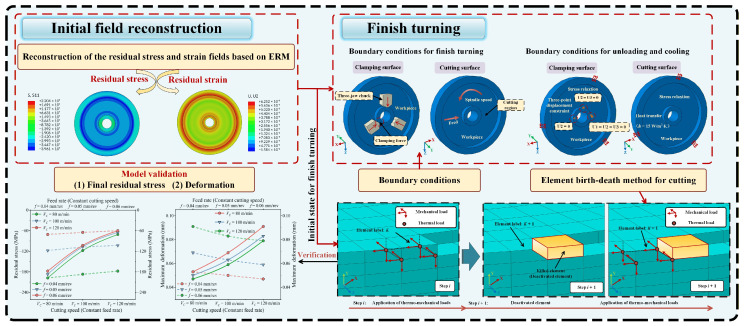
Construction of the prediction model for residual stress and deformation in finish turning of thin-web gears.

This study constructs a finish-turning-simulation model for thin-web gears in ABAQUS 2022 and proposes an initial field-reconstruction strategy to globally reconstruct the residual stress/strain field after carburizing–press quenching heat treatment, using it as the initial condition for the finish-turning model. During the cutting process, the element birth–death method is employed to simulate material removal, while thermo–mechanical loading (TML) is applied to model the thermo–mechanically coupled interaction between the workpiece and the cutting tool. The boundary conditions adopt a clamping method consistent with the experiments to obtain a more realistic loading state. After machining, the workpiece is unloaded and cooled to reach an equilibrium state after stress relaxation. Based on this model, this study further analyzes the effects of different cutting parameters and clamping forces on the FRS distribution and machining deformation of thin-web gears. The simulation workflow for finish turning of thin-web gears is shown in Figure 1.

### 2.1. Initial Residual Stress/Strain Field Reconstruction

Before establishing the finish-turning-simulation model, the residual stress/strain field generated by carburizing–press quenching was reconstructed and introduced as the initial state of the workpiece. This treatment allows the numerical model to start from the actual heat-treatment state rather than from an ideal stress-free and undeformed condition. The reconstruction was based on two groups of experimental data: the residual stress distribution measured via X-ray diffraction in the gear-web region and the deformation measured via coordinate measuring machine (CMM) and LOMM after heat treatment. These data were used together to characterize both the stress state and the macroscopic deformation response of the thin-web gear after heat treatment.

The reconstruction was carried out according to the eigenstrain reconstruction concept. In this method, the residual stress and deformation after heat treatment are regarded as the elastic response caused by the internal inelastic strain accumulated during carburizing, quenching, and unloading. The unknown inelastic strain field was represented by a set of smooth basis functions distributed along the radial and thickness directions of the gear web. Each basis function was individually introduced into an elastic finite element model to calculate the corresponding stress and displacement response. By comparing the calculated responses with the measured residual stress and deformation data, the contribution of each basis function was determined through a regularized inverse identification procedure. The final reconstructed field was then obtained by superposing these weighted basis responses and was mapped to the finish-turning finite element mesh as the predefined initial residual stress/strain field.

Several assumptions were adopted in the reconstruction process. First, after heat treatment, unloading, and cooling to room temperature, the thin-web gear was assumed to be in a self-equilibrated residual stress state. Second, considering the nearly axisymmetric geometry of the web and the constraint effect of the press-quenching die, the dominant variation of the initial residual field was assumed to occur in the radial–thickness plane. Third, the measured deformation after heat treatment was considered to be mainly caused by residual elastic strain release and heat-treatment-induced plastic strain accumulation. Fourth, during the reconstruction analysis, no cutting load or clamping load was applied. Only the minimum three-point displacement constraint was used to suppress rigid-body motion while avoiding artificial restriction of stress redistribution.

The accuracy of the reconstructed initial field may be affected by several factors, including the scatter of X-ray residual stress measurements, the alignment deviation of CMM and LOMM measurements, the limited number of measurement points, the truncation of basis functions, mesh interpolation error, and the smoothing effect introduced during inverse identification. Therefore, the reconstructed field should be understood as an experimentally constrained approximation of the actual heat-treatment state. Its reliability was further evaluated by comparing the reconstructed residual stress and deformation with the corresponding experimental measurements.

### 2.2. Finite Element Model, Thermo-Mechanical Loading, and Mesh Verification

**Figure 2:**
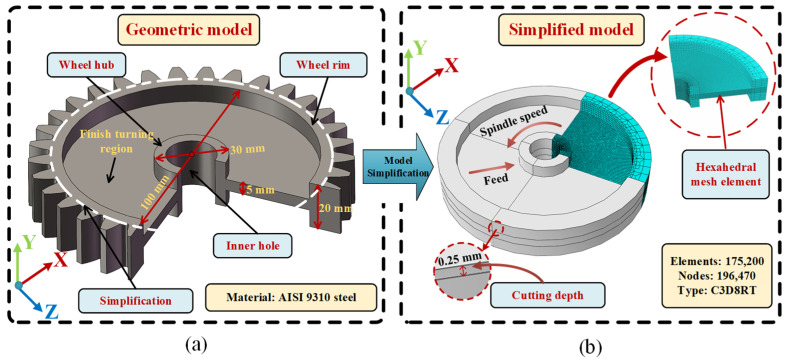
Geometric model and simplified model of thin-web gears: (**a**) geometric model; (**b**) finite element simplified model.

The geometric dimensions of the thin-web gear prior to finish turning are as follows: overall outer diameter of 120 mm, tooth width of 20 mm, wheel hub diameter of 30 mm, web diameter of 100 mm, and web thickness of 5 mm. The geometric model and the material used (AISI 9310 steel) are shown in Figure 2a. Before machining, the specimens undergo carburizing–press quenching heat treatment. Press quenching is a special quenching method in which a pressure die constrains key regions of the gear during quenching, thereby effectively suppressing workpiece deformation. The physical properties of AISI 9310 steel are listed in Table 1.

Based on the reconstructed initial field, a simulation analysis of finish turning for thin-web gears was conducted. Under the constant cutting condition of *V_C_* = 80 m/min, *f* = 0.04 mm/rev, and *a_p_* = 0.25 mm, five clamping-force levels (1200, 1350, 1500, 1650, and 1800 N) were first analyzed. This interval was selected from the anti-slippage requirement under the measured cutting force, the allowable loading range of the three-jaw chuck, and preliminary finish-turning observations. The lower bound was used to identify the onset of insufficient clamping, whereas the upper bound was used to evaluate deformation amplification caused by excessive constraint stress. A 150 N increment was adopted to capture the transition from unstable clamping to excessive bending deformation. After the feasible clamping interval was determined, the FRS distribution and deformation characteristics in the gear-web region were simulated under different cutting parameters (Table 2). Considering that finish turning only acts on the web region, the model was structurally simplified to ensure mesh accuracy and computational efficiency. The simplified finite element model is shown in Figure 2b. The model was meshed with C3D8RT elements, comprising 175,200 elements and 196,470 nodes. A mesh-sensitivity check was performed by comparing coarser and finer discretizations; the final mesh was selected after further refinement produced only marginal changes in the predicted maximum web deformation and near-surface FRS trend.

**Figure 3:**
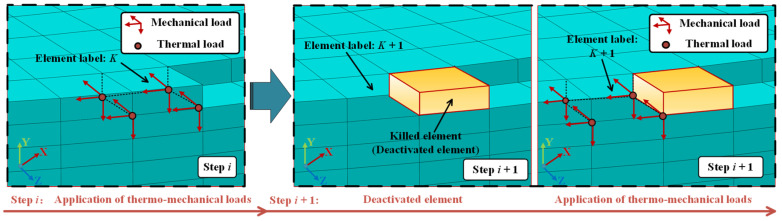
Application mechanism of thermo-mechanical loads in the element birth-and-death method.

**Figure 4:**
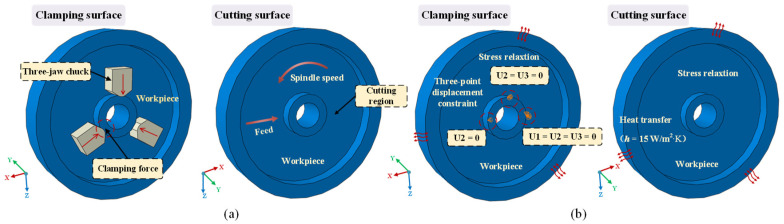
Boundary condition settings in the finish turning process of thin-web gears: (**a**) boundary conditions for finish turning; (**b**) boundary conditions for unloading and cooling. The arrows indicate the spindle rotation and feed directions.

Thin-web gears, as typical thin-walled components, have a radial dimension that is significantly larger than the axial dimension, resulting in a high aspect ratio. To capture the stress and temperature gradients along the thickness direction while maintaining acceptable computational cost, a multi-layer C3D8RT mesh and an element birth–death strategy were adopted instead of a fully dynamic chip-formation simulation. The thermo-mechanical loading (TML) applied in the model was obtained from the finish-turning experiments. The three-component cutting force measured by the Kistler dynamometer was transformed into the global x, y, and z directions and distributed to the nodes of the active element according to the current cutting position. The cutting temperature measured by the calibrated thermal imager was imposed as the corresponding thermal load on the same local contact zone. For the current material-removal element, denoted as element k, the mechanical and thermal loads were ramped to the measured values to represent the tool–workpiece interaction. In the next step, the TML on this element was removed and the element was deactivated to represent complete material removal. The TML was then transferred to the next element, denoted as element k + 1, as shown in Figure 3. This cyclic loading–unloading–deactivation–reloading sequence reproduces the progressive redistribution of IRS, MIRS, and clamping stress during finish turning. During machining, the three-jaw chuck load was applied to the clamping surface to reproduce the experimental constraint state (Figure 4a). After machining, the clamping force was released, convective heat transfer with h = 15 W/(m^2^·K) was applied until room temperature was reached, and three non-collinear displacement constraints were retained only to eliminate rigid-body motion and allow internal stress re-equilibration (Figure 4b).

## 3. Experimental Design and Validation

**Figure 5:**
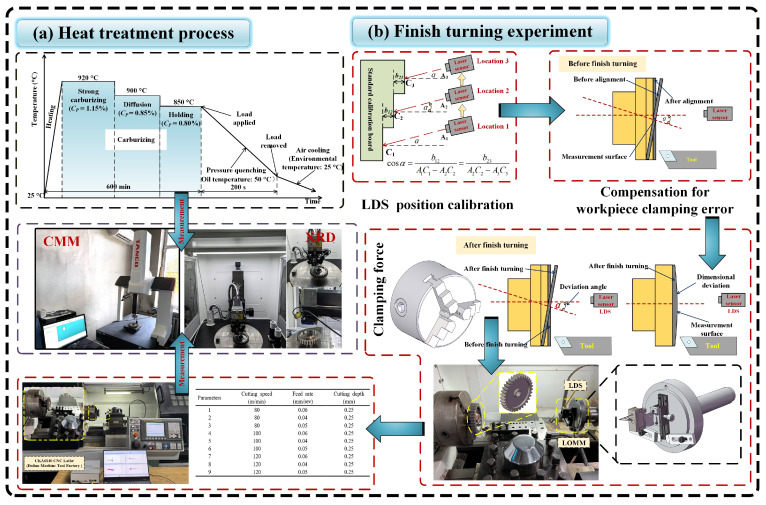
Finish turning and measurement experiments for thin-web gears.

To obtain the experimental data required for reconstructing the initial state in the finish turning simulation model, this study first measures the deformation and RS of thin-web gears after heat treatment. During the finish-turning experiments, in situ inspection of the workpiece is conducted based on the LOMM method. Meanwhile, by comparing the machining deformation results under different cutting parameters and clamping force conditions, the influence of these factors on web deformation is systematically analyzed. After finish turning, the FRS distribution and machining deformation of thin-web gears are measured and compared with the simulation predictions to validate the prediction accuracy of the constructed finish turning simulation model. The overall experimental workflow is shown in Figure 5.

### 3.1. Finish-Turning Experiments

**Figure 6:**
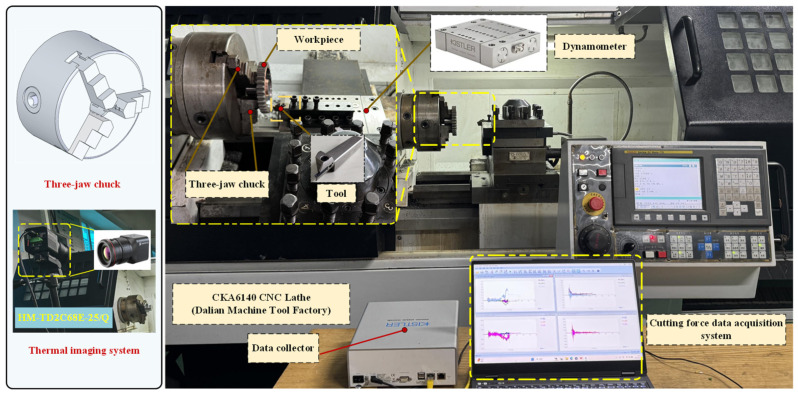
Experimental setup for finish turning of thin-web gears.

The finish-turning experiments of thin-web gears were conducted on a CKA6140 CNC lathe (Dalian Machine Tool Group, Dalian, China), with the experimental setup shown in Figure 6, and the cutting parameters are listed in Table 2. After heat treatment, the surface hardness of the web was approximately HRC 35–42. The cutting tool was a TiAlN-coated grooving insert (GTD300E030-MG-GM1230, TaeguTec, Daegu, Republic of Korea). Cutting force data were collected using a Kistler 9319 piezoelectric three-component dynamometer (Kistler Instrumente AG, Winterthur, Switzerland). To avoid transient entry and exit effects, the stable cutting segment was extracted, and its average value was used as the representative mechanical load for the simulation. The cutting temperature was measured using a HIKMICRO HM-TD2C68E-25/Q thermal imager (Hangzhou Hikmicro Sensing Technology Co., Ltd., Hangzhou, China). Before the experiments, the workpiece was preheated, and the thermal imager emissivity was calibrated using a thermocouple to reduce temperature measurement error. For each measurement location, three repeated measurements were performed. The mean value was used for comparison with the simulation, while the scatter among repeated measurements was used to evaluate measurement repeatability.

**Figure 7:**
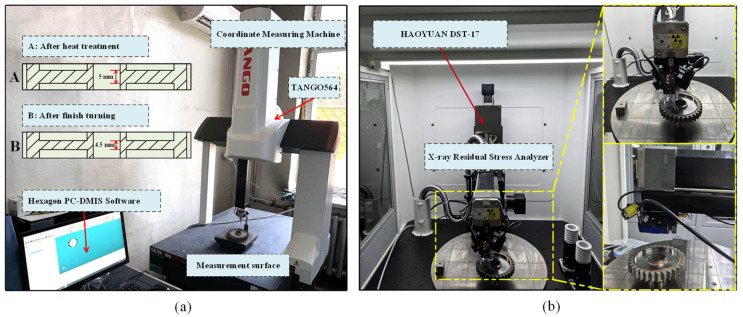
Deformation and residual stress measurement experiments for thin-web gears: (**a**) deformation measurement; (**b**) residual stress measurement.

Taking the web center as the coordinate origin, the deformation of thin-web gears after heat treatment and after finish turning was measured using a TANGO564 coordinate measuring machine (CMM) (Serein, Shenzhen, China), as shown in Figure 7a. RS was measured using a HAOYUAN DST-17 X-ray stress analyzer (Dandong Haoyuan Instrument Co., Ltd., Dandong, China) to obtain the RS distribution on the web after heat treatment and after finish turning, as shown in Figure 7b. To minimize measurement error and enhance result reliability, each measurement point was measured three times, and the average value was taken as the final result. The standard deviation of repeated measurements was used to estimate the measurement uncertainty. The above experimental data were used to validate the prediction accuracy of the finish-turning simulation model for both the FRS distribution and machining deformation. In addition, possible uncertainty sources, including workpiece-to-workpiece heat-treatment variation, tool wear, thermal drift, alignment error, XRD measurement scatter, and model simplifications, were considered in the discussion of result reliability.

### 3.2. Laser Displacement Sensor On-Machine Measurement Method and Application

**Figure 8:**
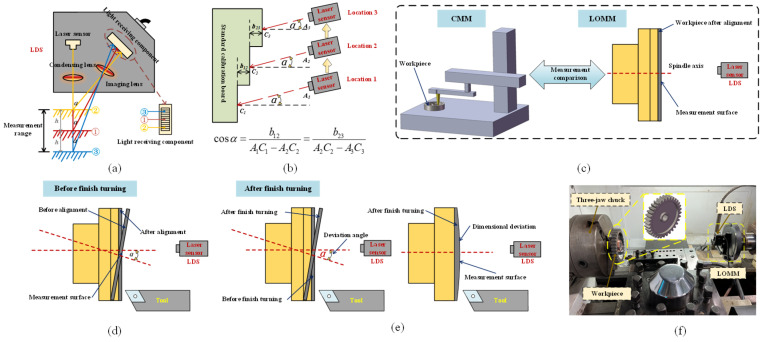
LOMM method and experimental setup: (**a**) principle of laser triangulation measurement, (**b**) LDS calibration method, (**c**) correction of workpiece pose deviation, (**d**) verification of LOMM measurement accuracy, (**e**) optimization method for the clamping force interval, (**f**) LOMM experimental setup.

This study proposes an LOMM method that enables in situ measurements of the workpiece by integrating a laser displacement sensor (LDS) into the lathe. The LDS is based on the laser triangulation principle, and its working principle is shown in Figure 8a. To ensure measurement accuracy, the LDS coordinate system was aligned with the machine-tool coordinate system before measurement. During calibration, a standard calibration board was measured at three circumferential positions to determine the sensor installation angle and spatial offset (Figure 8b). After calibration, LOMM was used to detect and correct the workpiece pose deviation before finish turning, as shown in Figure 8c. The corrected measurement coordinates were then used for post-machining deformation extraction and clamping-force interval optimization. The accuracy of LOMM was evaluated by comparing the same workpiece measured by CMM and LOMM (Figure 8d). The comparison included the mean deviation, maximum deviation, and repeatability deviation of repeated measurements. The measured difference between LOMM and CMM was approximately ±0.004 mm, indicating that the proposed method is sufficiently accurate for thin-web gear deformation monitoring. For clamping-force optimization, the initial 1200–1800 N range was selected according to the anti-slip requirement under measured cutting force, the operating capability of the three-jaw chuck, and preliminary cutting observations. After machining, the workpiece was inspected on-machine using LOMM (Figure 8e). Insufficient clamping was identified by slippage, chatter, and surface waviness, whereas excessive clamping was identified by increased bending deformation. Based on these criteria, the clamping-force interval was progressively revised and optimized.

## 4. Results and Discussion

### 4.1. Initial Field Reconstruction in Finish Turning

**Figure 9:**
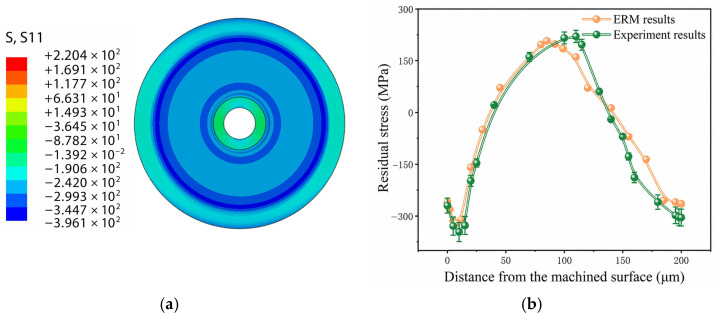
Reconstructed initial residual stress field and validation: (**a**) residual stress field reconstructed by ERM, (**b**) comparison of ERM and experimental results for the residual stress distribution along.

Based on the eigenstrain reconstruction method (ERM) proposed by Jun et al. [34], the global residual stress/strain field of thin-web gears after heat treatment was reconstructed, as shown in Figure 9a. A relatively high level of residual compressive stress appears on the inner and outer surfaces of the web. This is mainly attributed to the larger wall thickness at the junctions with the wheel hub and wheel rim, where thermal stress concentration becomes more pronounced. In contrast, the residual compressive stress levels in the surface layers of the wheel rim and wheel hub are relatively low because these regions are influenced by the flow channels of the press-quenching die, allowing the quenching medium to act directly on them. To validate the reliability of the reconstruction results, the reconstructed RS field was compared with the experimental measurements (Figure 9b). The reconstructed and measured results show consistent stress states and depth-wise trends.

**Figure 10:**
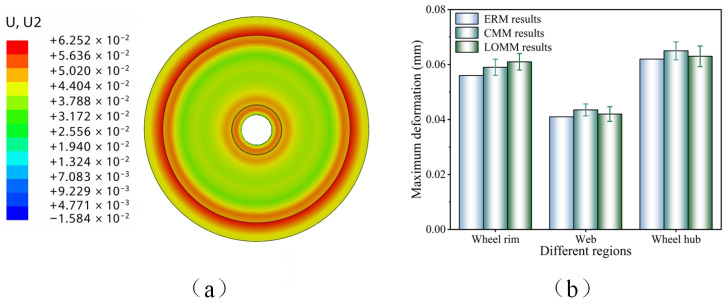
Reconstructed initial residual strain field and validation: (**a**) residual strain field reconstructed using the ERM, (**b**) comparison of the maximum deformation in different regions obtained using the ERM, CMM, and LOMM.

Figure 10a presents the reconstructed residual strain field, and Figure 10b compares the deformation of the wheel hub, wheel rim, and web with the experimental measurements. The relatively large deformation near the wheel hub, wheel rim, and the inner and outer web regions is related to the cooling-rate difference between the surface layer and the core during heat treatment, which produces a strong temperature gradient and non-uniform plastic strain. The remaining deviation between the reconstruction and experiment can be attributed to XRD measurement scatter, CMM/LOMM alignment error, basis-function truncation in the ERM, and interpolation error when mapping the reconstructed field to the finish-turning mesh. Overall, the agreement between the reconstructed and measured results indicates that the reconstructed initial field is reliable for subsequent finish-turning simulation.

To evaluate the measurement accuracy of the LOMM method, the thin-web gear after heat treatment was taken as the reference, and the measurement results obtained via CMM and LOMM were compared, as shown in Figure 10b. The two methods produce consistent deformation trends in the wheel rim, web, and wheel hub regions, with a deviation of approximately ±0.004 mm relative to the CMM measurements. This deviation is mainly caused by differences in the measurement environment and coordinate alignment, including ambient temperature fluctuation, lighting variation, the sensor installation angle, and workpiece re-clamping error. Because each point was measured repeatedly, the mean deviation and repeatability scatter were used to evaluate measurement reliability rather than relying on a single reading. The small discrepancy between the two methods confirms that LOMM can support initial pose correction, post-machining deformation extraction, and clamping-force interval optimization during finish turning.

### 4.2. Experimental Validation of Finish-Turning Simulation Results

#### 4.2.1. Clamping Force Interval Optimization

**Figure 11:**
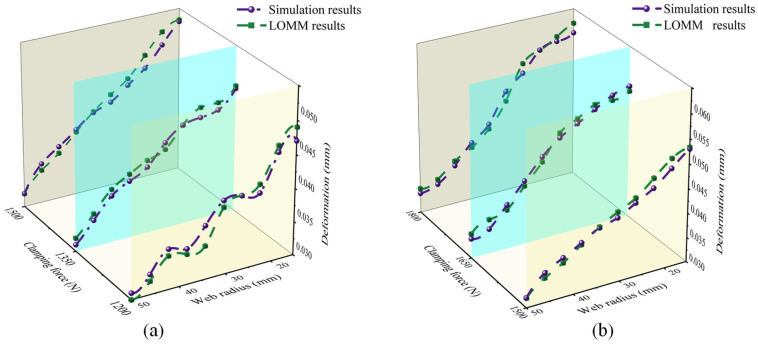
Comparison of the simulation predictions and LOMM measurements of web deformation under different clamping forces: (**a**) 1200–1500 N, (**b**) 1500–1800 N.

Clamping force is a critical parameter affecting cutting stability and deformation control in thin-walled components. To investigate its influence on thin-web gear deformation, comparative analyses between simulation and experiments were performed using the five clamping-force levels. The selected 1200–1800 N interval was not arbitrary: the lower level was used to test whether the frictional constraint at the jaw-workpiece interface was sufficient to resist the measured cutting load, while the upper level was used to evaluate whether excessive radial constraint would introduce additional bending deformation into the thin web. As shown in Figure 11, the simulation predictions agree well with the LOMM measurements, with a maximum relative error below 9.7%. When the clamping force is lower than 1350 N, the workpiece exhibits local slippage, chatter, and surface waviness during machining, indicating insufficient frictional constraint. When the clamping force exceeds 1650 N, cutting stability improves, but the bending deformation amplitude in the inner web region increases by approximately 11%. This occurs because the clamping-induced constraint stress is superposed with the IRS and MIRS during material removal, and the reduced web stiffness amplifies the resulting deformation. Therefore, clamping-force selection requires balancing the suppression of workpiece slippage with the avoidance of additional constraint-induced deformation. Based on the combined simulation and LOMM results, the optimal clamping-force interval under the current operating condition is determined to be 1350–1650 N.

#### 4.2.2. Final Residual Stress Analysis

**Figure 12:**
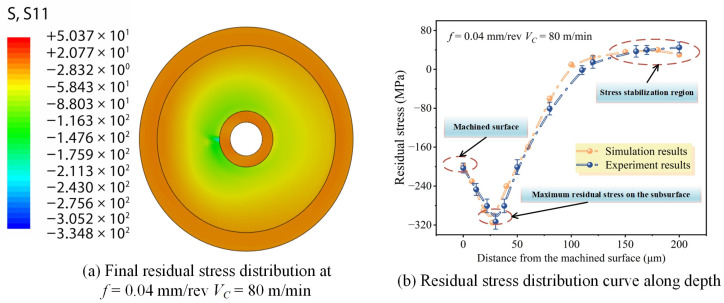
Simulation predictions and experimental validation of FRS in the thin-web gear after finish turning: (**a**) simulated FRS prediction results, (**b**) comparison between simulated and experimental FRS along the depth direction.

Figure 12a shows the simulated FRS distribution in a thin-web gear after finish turning under *f* = 0.04 mm/rev, *V_C_* = 80 m/min, and a clamping force of 1500 N. Figure 12b presents the FRS-distribution curves within a depth range of 200 μm beneath the machined surface. The comparison between the simulation and experiment indicates that the prediction model reproduces both the stress distribution trend and the surface stress state. The web surface exhibits residual compressive stress, and the peak compressive stress appears approximately within the 30–40 μm subsurface region. This feature is governed by the interaction between the inherited IRS and the MIRS generated by TML. During finish turning, the mechanical load and thermal gradient generate near-surface plastic strain, whereas the inherited heat-treatment IRS provides a pre-existing compressive/tensile stress gradient. Material removal breaks the original self-equilibrium of the heat-treatment field, causing stress release and redistribution. If the IRS after heat treatment is neglected, the calculated surface RS shifts from compressive stress to weak tensile stress, leading to a clear deviation from the experimental results. These results demonstrate that introducing the global residual stress/strain field after heat treatment into the finish-turning simulation model is essential for accurately predicting FRS evolution.

**Figure 13:**
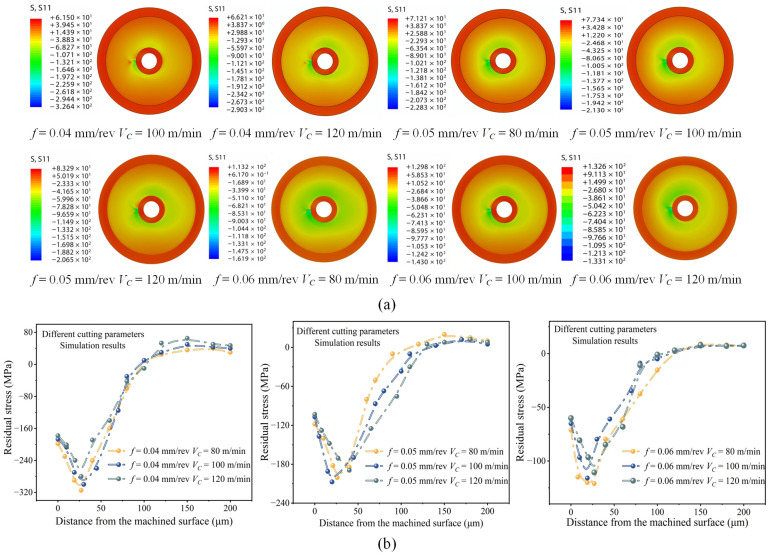
Simulated FRS results of thin-web gears after finish turning: (**a**) simulated FRS for different cutting parameters, (**b**) FRS distribution curves along the depth direction for different cutting parameters.

Figure 13a presents the FRS distribution results of thin-web gears after finish turning predicted by the simulation model under different cutting parameters. Stress concentration mainly occurs at the junction between the gear web and the wheel hub. This phenomenon is caused by the combined effects of machining sequence, stiffness discontinuity, and residual-stress redistribution. When the outer-ring material of the gear web is removed first, the unmachined inner region can still provide structural support. As machining progresses toward the wheel hub, the remaining web thickness decreases, the local bending stiffness is reduced, and the hub-web transition becomes a sensitive region for stress concentration. Figure 13b shows the FRS variation within a depth range of 200 μm from the machined surface. The FRS exhibits a typical spoon-shaped distribution along the depth, resulting from the superposition of mechanical extrusion, thermal softening, plastic strain recovery, and inherited IRS release. The web surface mainly shows residual compressive stress. With an increasing depth, the compressive stress amplitude first increases and then decreases, reaching a peak at approximately 30–40 μm below the machined surface, with a maximum compressive stress of −315 MPa. Because the finish-turning parameters are relatively small, the near-surface layer remains dominated by compressive stress. At greater depths, the stress state gradually transfers from compressive to tensile and tends to stabilize at approximately 100 μm below the machined surface. Increasing the cutting speed and feed rate reduces the residual compressive stress level on the surface to different extents because the stronger thermo-mechanical action weakens the inherited compressive state and promotes stress redistribution.

#### 4.2.3. Machining Deformation Analysis

**Figure 14:**
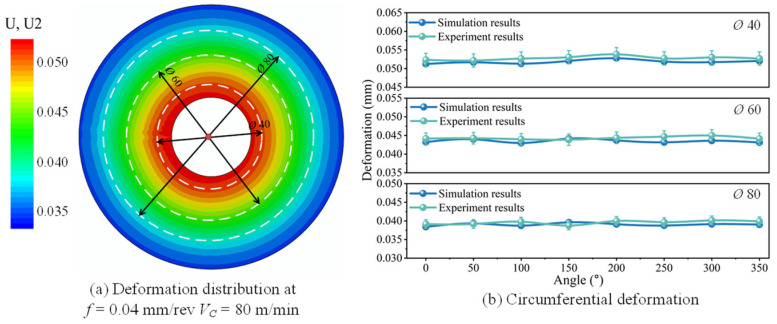
Simulation prediction and experimental validation of machining deformation after finish turning: (**a**) simulated machining deformation results, (**b**) comparison of simulated and experimental circumferential deformation at different diameters.

After finish turning, the simulated machining deformation of the web region is shown in Figure 14a. The web deformation gradually increases along the radial direction from the outer side to the inner side, and the maximum deformation is concentrated in the inner web region. This deformation pattern is caused by the joint action of material-removal-induced stiffness reduction, inherited IRS release, MIRS formation, and clamping-stress relaxation. After the outer-side material is removed, the original stress equilibrium of the web is disturbed. The remaining thin web has limited bending stiffness and is therefore more sensitive to the residual stress gradient. Near the web-hub junction, the abrupt change in section thickness restricts free deformation and produces local stress concentration, further amplifying the deformation of the inner web region. To validate the prediction accuracy of the simulation model, deformation values on the circumferences with diameters of 40 mm, 60 mm, and 80 mm were extracted and compared with the experimental measurements, as shown in Figure 14b. The results indicate that the deformation amplitude increases radially toward the inner side, and the simulation results agree well with the experimental measurements. This demonstrates that the constructed simulation model can reliably reflect the deformation characteristics of the web structure at different radial positions.

**Figure 15:**
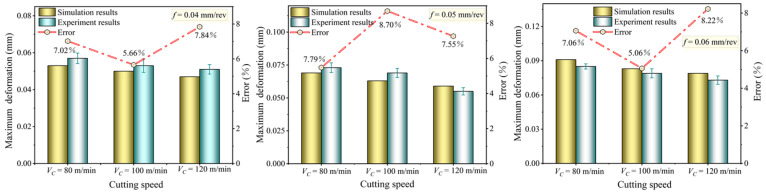
Comparison of the simulated and experimental maximum deformation of thin-web gears after finish turning.

Figure 15 compares the simulation predictions and experimental measurements of the maximum deformation in the web region. The maximum relative error is below 9%, and the average error is below 8%, indicating a low overall error level. The remaining error is mainly associated with four factors: (i) the simulation does not explicitly update cutting load evolution induced by tool wear; (ii) the element birth–death method neglects detailed thermal conduction between the cutting tool and workpiece; (iii) the experimentally reconstructed initial field contains measurement and interpolation uncertainty; and (iv) small differences in clamping contact and workpiece alignment exist between the model and the experiment. Despite these simplifications, the constructed finish-turning-simulation model significantly improves the prediction accuracy of FRS and machining deformation by considering both the initial heat-treatment state and experimentally consistent clamping constraints.

#### 4.2.4. Effects of Cutting Parameters on Final Residual Stress and Maximum Deformation

**Figure 16:**
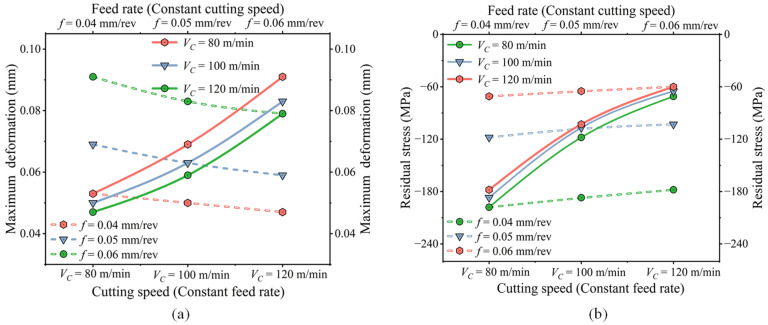
Effects of cutting parameters on the maximum deformation and FRS of thin-web gears: (**a**) effects of cutting parameters on maximum deformation; (**b**) effects of cutting parameters on FRS.

Figure 16a,b compare the FRS and maximum deformation on the machined surface of the web under different cutting parameters. The results indicate that both the tensile stress and the maximum deformation on the web surface increase as the feed rate increases. This is mainly because an increase in feed rate significantly increases the cutting force and cutting temperature, intensifying plastic deformation in the surface layer and thereby generating a higher tensile stress gradient, which aggravates the bending deformation of the workpiece. Therefore, during finish turning, a smaller feed rate should be preferably selected to effectively control machining deformation and reduce the tensile stress level on the machined surface. In contrast, the effect of the cutting speed on machining deformation and FRS is relatively weak. Increasing the cutting speed shortens the contact time between the tool and the workpiece, allowing most of the heat to be rapidly carried away by chips. This reduces the cutting temperature at the surface and consequently reduces the formation of tensile stress and machining deformation. In summary, for the finish turning of thin-web gears, a higher cutting speed combined with a smaller feed rate is recommended to effectively reduce machining deformation and surface residual tensile stress, thereby improving machining quality and service performance.

### 4.3. Repeatability and Uncertainty Analysis

The repeatability and uncertainty of the results were evaluated from both the experimental and numerical perspectives. Experimentally, each CMM, XRD, and LOMM measurement point was measured three times, and the average value was used for comparison with the simulation. The scatter of repeated measurements was used to estimate measurement repeatability. The main experimental uncertainty sources include specimen-to-specimen variation after carburizing–press quenching, tool wear during finish turning, temperature drift, sensor alignment error, re-clamping error, and XRD measurement scatter. Numerically, uncertainty mainly comes from the reconstructed initial field, mesh interpolation, simplification of tool-workpiece contact into equivalent TML, omission of detailed tool heat conduction, and the idealization of the three-jaw contact area. Because repeated machining tests for every cutting-parameter combination are costly for heat-treated thin-web gears, the current validation focuses on trend consistency, the error level, and agreement under key operating conditions. Future work should increase the number of repeated machining specimens and add confidence intervals to further improve statistical robustness.

## 5. Conclusions

This study establishes an initial-field-driven simulation prediction model for the finish turning of aerospace thin-web gears. The global residual stress/strain field after carburizing–press quenching is reconstructed and inherited as the initial condition of finish turning, while clamping constraints consistent with the experimental three-jaw chuck are introduced to replace idealized fixed boundaries. The LOMM method is further used for workpiece pose correction, deformation measurements, and clamping-force interval optimization. Based on the revised modeling, experimental validation, and uncertainty analysis, the following conclusions are drawn:

(1) By reconstructing the post-heat-treatment initial field of thin-web gears and introducing it into the finish-turning simulation model, the actual IRS/strain state after carburizing–press quenching is inherited in the machining stage. Combined with experimentally consistent clamping constraints, this strategy reduces the deviation caused by stress-free initial assumptions and idealized fixed boundaries, keeping the FRS and deformation prediction errors generally within 10%.
(2) The LOMM method enables initial pose correction, post-machining deformation extraction, and clamping-force interval optimization. Compared with CMM measurements, the LOMM results show a small deviation of approximately ±0.004 mm, confirming that the method can provide reliable on-machine data for simulation validation and machining-state correction.
(3) When the clamping force is insufficient, the frictional constraint at the clamping interface is weakened and has difficulty resisting the cutting load, readily causing microslip of the workpiece and triggering cutting chatter. When the clamping force is excessive, it introduces constraint stress and is nonlinearly coupled with MIRS/IRS, thereby aggravating flexural deformation of the web. Based on LOMM feedback, the clamping force interval is corrected to strike a balance between deformation control and cutting stability, thereby improving machining quality and process robustness.
(4) For the finish turning of thin-web gears with a diameter of 120 mm and a web thickness of 5 mm, the feed rate is the key parameter affecting the FRS level and machining deformation, whereas the effect of the cutting speed is relatively weak. As the feed rate increases, the compressive residual stress on the machined surface gradually decreases and tends to shift toward a tensile state, while deformation on the inner side of the web is aggravated. Using a smaller feed rate and appropriately increasing cutting speed helps suppress the residual tensile stress level on the machined surface and control machining deformation.

## Figures and Tables

**Table 1:** Physical properties of the AISI 9310 steel.

Material Properties	Value
Density (g/cm^3^)	7.691
Young’s modulus (GPa)	221
Poisson’s ratio	0.29
Thermal conductivity (W/m·K)	24.309
Specific heat (J/kg·K)	642
Thermal expansion (°C^−1^)	12.648 × 10^−6^

**Table 2:** Cutting parameters for the finish turning experiments.

Parameters	Cutting Speed *V_C_* (m/min)	Feed Rate *f* (mm/rev)	Cutting Depth *a_p_* (mm)
1	80	0.06	0.25
2	80	0.04	0.25
3	80	0.05	0.25
4	100	0.06	0.25
5	100	0.04	0.25
6	100	0.05	0.25
7	120	0.06	0.25
8	120	0.04	0.25
9	120	0.05	0.25

## Data Availability

The original contributions presented in this study are included in the article. Further inquiries can be directed to the corresponding author.
